# Combination of Intravesical Chemotherapy and Bacillus Calmette–Guerin Versus Bacillus Calmette–Guerin Monotherapy in Intermediate- and High-risk Nonmuscle Invasive Bladder Cancer: A Systematic Review and Meta-analysis

**DOI:** 10.1097/MD.0000000000002572

**Published:** 2016-01-22

**Authors:** Jianfeng Cui, Wenbo Wang, Shouzhen Chen, Pengxiang Chen, Yue Yang, Yunliang Guo, Yaofeng Zhu, Fan Chen, Benkang Shi

**Affiliations:** From the Department of Urology, Qilu Hospital of Shandong University (JC, SC, YY, YZ, FC, BS); Department of Endocrinology and Metabolism, Shandong Provincial Hospital affiliated to Shandong University (WW); Department of Radiation Oncology, Qilu Hospital of Shandong University (PC); and Department of Neurology, Shandong Provincial Hospital Affiliated to Shandong University, Jinan, People's Republic of China (YG).

## Abstract

Urothelial carcinoma of the bladder has become a major cause of morbidity, mortality, and health-related costs. There is still no standard instillation therapy against bladder cancer. A meta-analysis was conducted to evaluate the efficacy and toxicity of adding chemotherapy to Bacillus Calmette–Guerin (BCG) in intermediate- and high-risk nonmuscle invasive bladder cancer (NMIBC).

All randomized controlled trials (RCTs) that evaluated the efficacy of combination therapy and BCG monotherapy for intermediate- and high-risk NMIBC were comprehensively searched. Relevant databases, including PubMed, Embase, Cochrane Central Register of Controlled trials databases, and American Society of Clinical Oncology (http://www.asco.org/ASCO), the clinical trial registration website (ClinicalTrials.gov), and relevant trials from the references of selected studies were searched from initial state up to June 6, 2015. Random-effects model was used to estimate hazard ratios (HRs) statistics. All statistical analyses were performed by STATA (version 13.0, College Station, TX).

Seven studies, including 1373 patients with intermediate- and high-risk NMIBC, were identified. For disease-free survival, the pooled HRs from all studies was 0.69 (95% confidence interval [CI], 0.48–1.00; *P* = 0.048). The disease-free survival benefit was more apparent among patients with intermediate-risk NMIBC (*P* = 0.002) or Ta/T1 with/without carcinoma in situ (*P* < 0.01). In subgroup analysis, a significant reduction in recurrence was found in studies that explored the influence of a perioperative single dose instillation compared with delayed BCG monotherapy (HR = 0.60; 95% CI, 0.38–0.92; *P* = 0.021). No significant difference was found for progression-free survival (HR = 0.78; 95% CI, 0.43–1.44; *P* = 0.435).

Patients with intermediate- and high-risk NMIBC who underwent combination therapy achieved lower rates of recurrence than those who underwent BCG therapy alone. No difference in progression-free survival was found between the 2 different therapy schedules. Better efficacy for a perioperative single dose instillation compared with delayed BCG monotherapy was found in this meta-analysis.

## INTRODUCTION

Bladder cancer has become the fourth most common cancer, with an incidence of approximately 7%, and the eighth most common cause of mortality in men (approximately 4%) according to the newly published global cancer statistics.^1^ Among the urogenital system cancers, bladder cancer is ranked second both in incidence and mortality. Approximately, 74,000 new bladder cancer cases are expected with 16,000 estimated deaths for both sexes in the United States.^1^ Approximately, 75% of patients with bladder cancer are diagnosed with nonmuscle invasive bladder cancer (NMIBC).^2^ Nonmuscle invasive bladder cancer includes Ta (noninvasive papillary carcinoma), T1 (tumor invades subepithelial connective tissue), and carcinoma in situ (CIS: “flat tumor”). The cancer is graded using a combination of cystoscopy, urinary cytology, and multiple bladder biopsies. According to the International Bladder Cancer Group review,^3^ the definitions of risk are as follows: low risk, solitary, primary low-grade (Ta) tumor (these tumors have a low risk of recurrence and progression); intermediate risk, multiple or recurrent low-grade tumors (these tumors have an intermediate to high risk of recurrence, but low to intermediate risk of progression); and high risk, any T1 and/or high-grade/G3 and/or CIS (these tumors have a high risk of recurrence and progression, with progression being the primary concern).

Bacillus Calmette–Guerin's anti-tumor effect was first described by Morales.^4^ Since then, the superiority of Bacillus Calmette–Guerin (BCG) for the prevention of recurrence and progression in intermediate- and high-risk NIMBCs compared with a combination of epirubicin and interferon,^5^ mitomycin C (MMC),^6^ or epirubicin^7^ has been confirmed in a number of studies. Although BCG demonstrated strong efficacy, a large number of patients still suffered from recurrence and progression to muscle-invasive bladder cancer.^8^ The cost increases concomitant with disease recurrence and progression, and therefore the patient's quality of life could be affected. Therefore, it is critical to explore new strategies to lower the recurrence and progression rates. Several studies demonstrated that combination therapy had better efficacy than BCG monotherapy, but these findings remained controversial. At present, whether the combination of intravesical chemotherapy and BCG has better efficacy and less toxicity compared with BCG monotherapy remains unknown. In addition, there is still a controversy involving that treatment is better, perioperative single-dose instillation or delayed BCG monotherapy.

In this study, we conducted a meta-analysis to evaluate the efficacy and toxicity of adding chemotherapy to BCG in intermediate- and high-risk NMIBC. The impact of other potential covariates (i.e., the chemotherapy agent and tumor stage) was also investigated.

## METHODS

### Ethnic Statement

Ethnic approval is not necessary for this meta-analysis.

### Selection Criteria

Studies that were published in English were selected if they met the following criteria: all patients were pathologically diagnosed with NIMBC; all patients had intermediate- or high-risk NIMBC (whether primary or not); all patients underwent transurethral resection of the bladder tumor; the interventions were intravescial chemotherapy plus BCG versus monotherapy; the intravescial chemotherapy consisted of a perioperative single dose instillation, and the chemotherapy agents were MMC and epirubicin; the study design was a randomized controlled trial (RCT); and studies in patients who received neoadjuvant treatment or intravenous chemotherapeutic or immunomodulatory agents were excluded.

### Search Strategy

To identify studies that met the above selection criteria, we searched the PubMed, Embase and the Cochrane Library, American Society of Clinical Oncology (http://www.asco.org/ASCO), the clinical trial registration website (ClinicalTrials.gov) databases and relevant trials from the references of selected studies (up to June 6, 2015). In addition, we manually searched for potentially relevant trials from the references of selected studies. The search strategy was followed using all possible combinations of the medical subject headings or nonmedical subject heading terms: MMC, mitocin C, NSC-26980, ametycine, mutamycin, and MMC; epirubicin, 4’-epidoxorubicin, and NSC-256942; intravescial chemotherapy; BCG; and bladder cancer, bladder carcinoma, bladder neoplasm, bladder tumor, and urinary bladder cancer. Each search strategy was conducted in each database.

### Data Extraction

Two reviewers (JFC and WBW) independently assessed all eligible publications. Disagreements were resolved by discussion with a third reviewer (SZC). Data from all full-text studies that met the selection criteria were independently extracted by each reviewer using a standardized extraction form. Data extracted from the studies included details on the author name, publication year, country, study period, number of patients, number of sexs, age, duration of follow-up, predominant risk factor in each study, therapy schedule, randomized, and event numbers of the combination and monotherapy group, and results (hazard ratios [HRs], 95% confidence intervals [CIs], and *P* values).

### Outcome Measures

The primary outcome measure in this meta-analysis was disease-free survival (DFS), that was defined as, the time from randomization to the first cystoscopy noting recurrence, and perioperative single dose instillation is defined as a single instillation finished within 6 hours of surgery. Delayed BCG monotherapy is defined as subsequent weekly BCG instillations finished at least 2 weeks after surgery. The secondary outcome measure was progression-free survival (PFS), defined as the time from randomization to a T2 or higher tumor or metastatic disease. Toxicity was also included as an outcome measure.

### Statistical Analysis

Differences were expressed as HRs with 95% CIs for the primary outcome and secondary outcome. An HR < 1 indicated an advantage of combination over monotherapy or a perioperative single dose instillation over no therapy. Hazard ratios included in the studies were extracted directly. For studies that did not provide HRs and CIs but included Kaplan–Meier log-rank tests or Wilcoxon *P* values, we used the methods reported by Parmar et al,^9^ Williamcon et al,^10^ and Tierney et al^11^ to estimate the HRs and 95% CIs. Heterogeneity across trials was quantified using the I^2^ statistic, that is a standardized measure of inconsistency, and the χ^2^ (Cochrane Q statistic) test. Trials with an I^2^ statistic more than 40% and a *P* value <0.1 for the χ^2^ test had a high level of heterogeneity. A random-effects model was used to pool estimates regardless of high or low levels of heterogeneity because we believed that this approach was more effective because of the heterogeneous nature of the treatment schedules between studies.

Patient characteristics, treatment schedules, and other confounding factors were not consistent between studies. Therefore, there was a significant advantage of a random-effects model compared with a fixed-effects model in accounting for heterogeneity between studies.^12^ Subgroup analyses were planned to assess the effect of different chemotherapy agents, tumor stages, and different levels of risk. A *P* value <0.05 was affirmed as statistically significant. All statistical analyses were performed by STATA (version 13.0, College Station, TX).

### Quality Assessment

The methodological quality of each RCT was evaluated using Cochrane collaboration's tool (version 5.2, The Nordic Cochrane Centre, The Cochrane Collaboration, USA),^13^ that is recommended to assess the risk of bias. This tool included 7 aspects to provide a qualification of the risk of bias: random sequence generation, allocation concealment, blinding of participants and personnel, blinding of outcome assessment, incomplete outcome data, selective outcome reporting, and other bias. The 2 reviewers gave each aspect a high, low, or unclear risk of bias. Disagreements were resolved through discussion.

### Level of Evidence

We used the GRADE (grading of recommendation, assessment, development, and evaluation) method^14^ to evaluate the strength of the evidence, including 5 downgrade qualities of evidence and 3 upgrade qualities of evidence. Moreover, the use of the GRADEprofiler (version3.6, GRADE Working Group) enabled the creation of an evidence profile.

The level of evidence in the GRADE system included: high quality, further research is very unlikely to change our confidence in the estimate of effect; moderate quality, further research is likely to have an important impact on our confidence in the estimate of effect and may change the estimate; low quality, further research is likely to have an important impact on our confidence in the estimate of effect and is likely to change the estimate; and very low quality, very uncertain about the estimate.

## RESULTS

**FIGURE 1:**
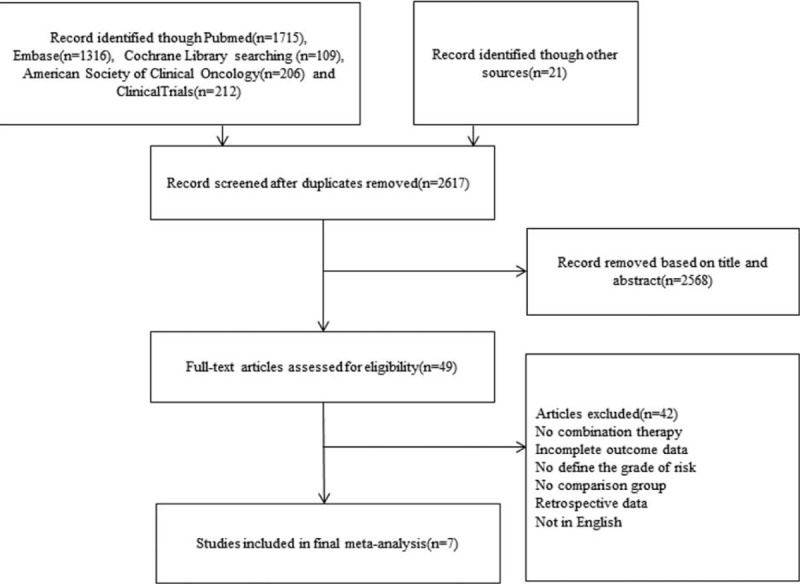
Selecting the flowchart for the inclusion of studies in the meta-analysis.

After removing 962 duplicates, 2617 potential studies were identified through reviewing abstracts and articles. A total of 42 studies were excluded for the following reasons: a lack of combination therapy; incomplete outcome data; no definition of the grade of risk; no comparison group; retrospective data; or not in English. The final set of eligible studies included 7 studies^15–21^ published from 1999 to 2014. The selection strategy was shown in Figure 1. The characteristics of the 7 included studies were summarized in Table 1. A total of 1373 patients were included in this meta-analysis. Of these patients, 702 patients were treated with combination therapy, and 671 underwent BCG monotherapy. Two studies^17,18^ included patients with CIS alone, 5 studies^15–19^ compared the efficacy between MMC + BCG and BCG monotherapy, and 2 studies^20,21^ compared the efficacy between epirubicin + BCG and BCG monotherapy. In addition, 2 studies^19,20^ investigated the efficacy of perioperative chemotherapeutic agent instillation compared with delayed BCG monotherapy, and 5 studies^16,18–21^ compared the toxicity between combination therapy and BCG monotherapy.

**TABLE 1:**
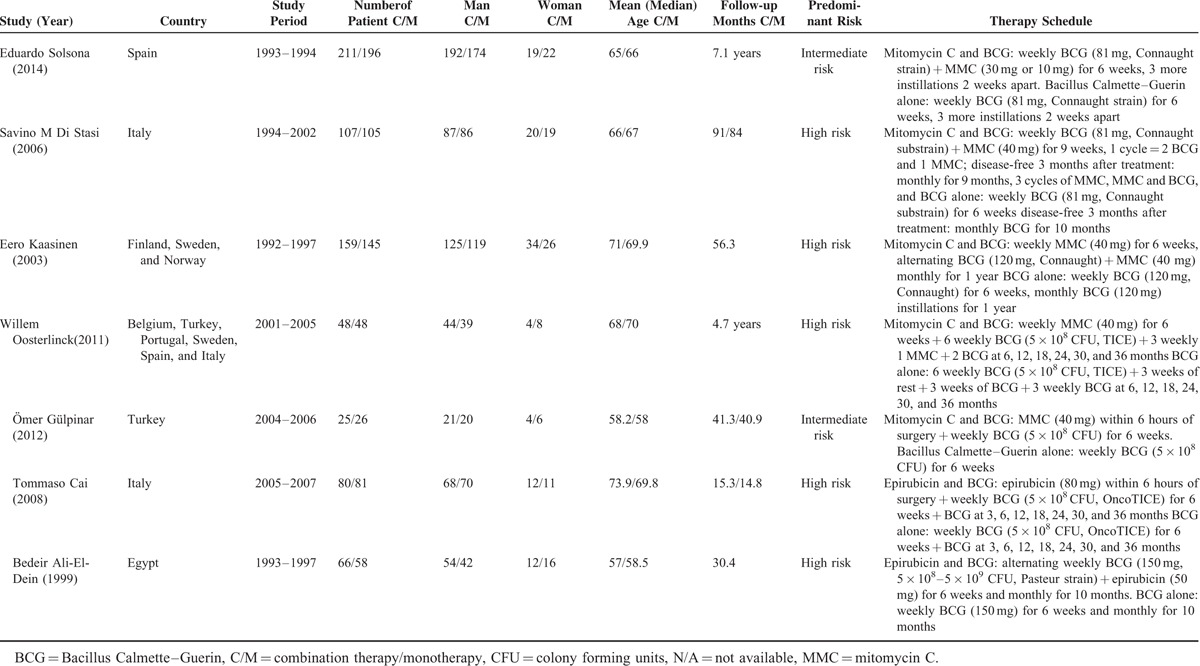
Characteristics of the Included Studies

### Effect of Interventions on the Primary Outcome Measure

**FIGURE 2:**
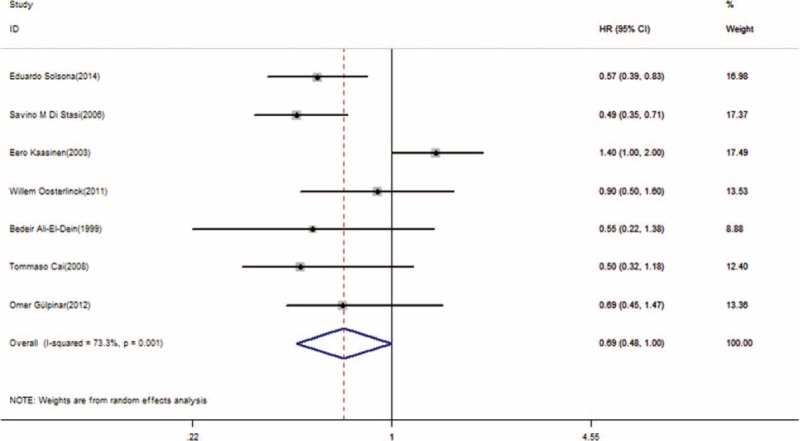
Forest plot of disease-free survival across all 7 studies. HR = hazard ratio, CI = confidence interval.

**FIGURE 3:**
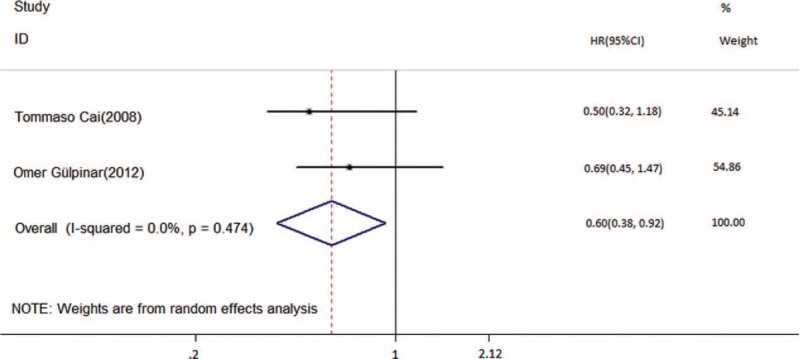
Forest plot of disease-free survival across 2 studies for perioperative instillation or not. HR = hazard ratio, CI = confidence interval, CIS = carcinoma *in situ*.

Disease-free survival was the primary outcome measure in this meta-analysis. Using a random-effects model, 5 multivariate HRs and CIs were directly extracted from the studies, and 2 HRs and CIs were calculated using the method mentioned before. The pooled HR was 0.69 (95% CI, 0.48–1.00; *P* = 0.048, Figure 2). This result represented a significantly better DFS (31% relative decrease in the risk of recurrence) in patients treated with combination therapy, although substantial heterogeneity existed (I^2^ = 73.3%; *P* = 0.001, Figure 2). The pooled HR from 2 studies revealed that 1 perioperative instillation significantly improved the efficacy of delayed BCG monotherapy in DFS (HR = 0.6; 95% CI, 0.38–0.92; *P* = 0.021, Figure 3) without heterogeneity.

**FIGURE 4:**
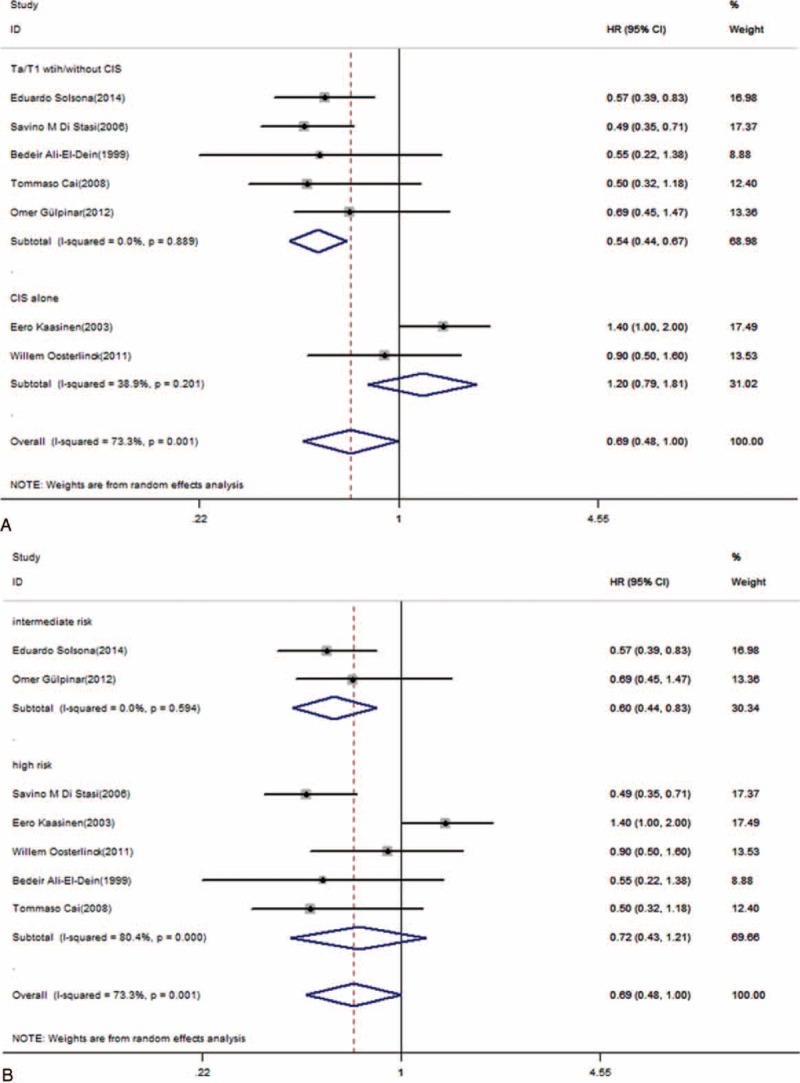
A, Forest plots of disease-free survival for different tumor stages. B, Different tumor risks. HR = hazard ratio, CI = confidence interval.

**FIGURE 5:**
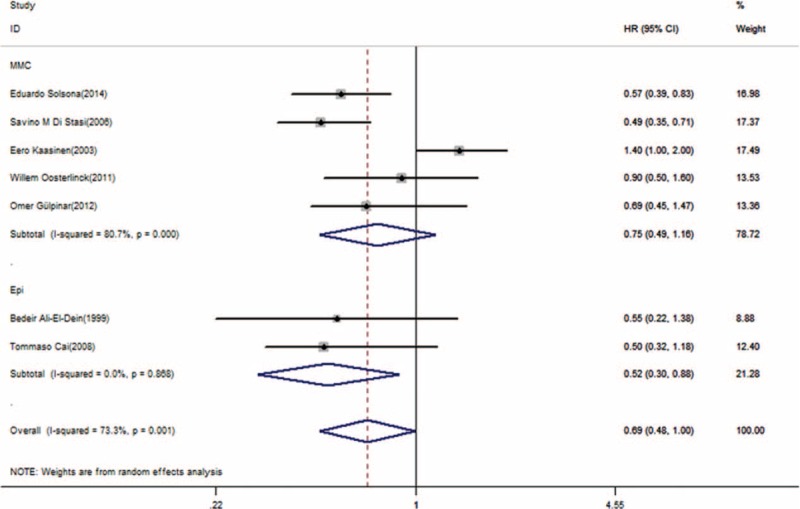
Forest plot of disease-free survival for MMC and epirubicin. HR = hazard ratio, CI = confidence interval, MMC = mitomycin, Epi = epirubicin.

The subgroup analyses were shown in Figures 4 and 5. The heterogeneity was explained by the tumor stage. Patients with Ta/T1 with (or without) the CIS stage benefited from combination therapy (HR = 0.54; 95% CI, 0.44–0.67; *P* = 0, heterogeneity *P* = 0.889, I^2^ = 0%, Figure 4), whereas patients with CIS alone did not gain any benefit from combination therapy (HR = 1.20; 95% CI, 0.79–1.81; *P* = 0.395, Figure 4). Carcinoma in situ is different from the Ta/T1 stage. Patients treated with BCG + MMC did not exhibit a significant difference compared with patients treated with BCG alone in terms of DFS (HR = 0.75; 95% CI, 0.49–1.16; *P* = 0.198, Figure 5), but there was a significant difference in DFS between BCG + epirubicin combination therapy and BCG monotherapy (HR = 0.52; 95% CI, 0.30–0.88; *P* = 0.015, Figure 5). The combination therapy had a more significant effect on reducing the recurrence rate of intermediate-risk NMIBC (HR = 0.6; 95% CI, 0.44–0.83; *P* = 0.002, Figure 4), but did not have a significant benefit for high-risk NMIBC (HR = 0.72; 95% CI, 0.43–1.21; *P* = 0.215, Figure 4).

### Effect of Interventions on the Secondary Outcome Measure

**FIGURE 6:**
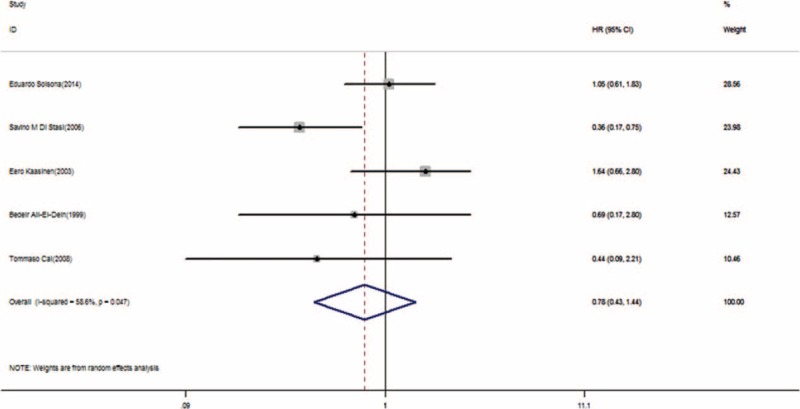
Forest plot of progression-free survival across 5 studies. HR = hazard ratio, CI = confidence interval.

Progression-free survival was the secondary outcome measure in this meta-analysis. A total of 5 studies were involved in this meta-analysis on PFS. This outcome measure should be interpreted with caution because of the low rate of progression. The outcome measure showed no significant effect on progression, with a pooled HR of 0.78 (95% CI, 0.43–1.44; *P* = 0.435, Figure 6). Substantial heterogeneity (I^2^ = 58.6%) was also observed.

**FIGURE 7:**
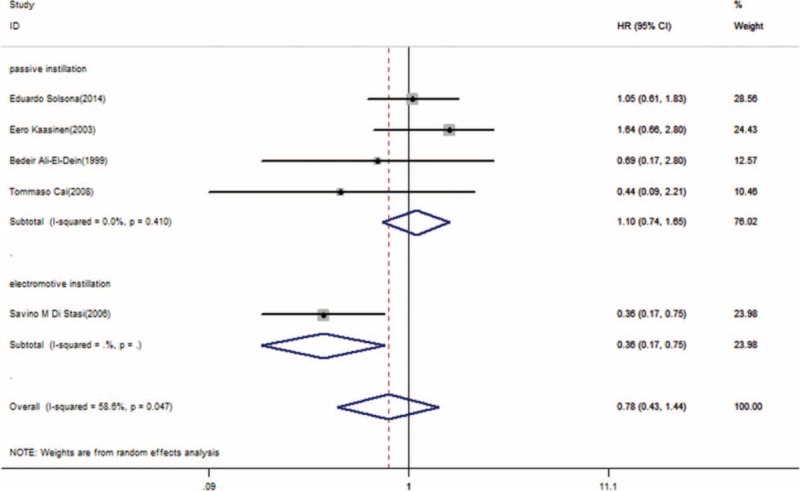
Forest plot of progression-free survival for different methods of instillation. HR = hazard ratio, CI = confidence interval.

The subgroup analyses were shown in Figure 7. We analyzed some potential reasons for heterogeneity in PFS, such as tumor risk, chemotherapy agents, etc. One potential explanation for this heterogeneity was the electromotive MMC from Di Stasi et al.^16^ No significant benefit was detected in patients who received passive instillation (HR = 1.10; 95% CI, 0.74–1.65; *P* = 0.635, heterogeneity *P* = 0.41; I^2^ = 0%).

### Effect of Interventions on Toxicity

Toxicity data could be extracted from 5 of the studies.^16,18–21^Table 2 showed the numbers and percentages of the primary side effects. A total of 644 patients were included in this analysis. The most common side effect in intravescial instillation was cystitis in this meta-analysis. In the combination therapy group, 25.5% of patients had symptoms of cystitis, compared with 30.2% of patients in the BCG monotherapy group (*P* > 0.1). The other side effects were slightly less common in the combination therapy group, but these differences did not reach statistical significance. One study^15^ reported that combination therapy was more toxic than BCG monotherapy despite decreasing the MMC from 30 mg to 10 mg. Another study^17^ showed that combination therapy was significantly better tolerated than BCG monotherapy. Cai et al^20^ reported that local toxicity decreased after antibiotic treatment. Colombel et al^22^ verified this conclusion.

**TABLE 2:**
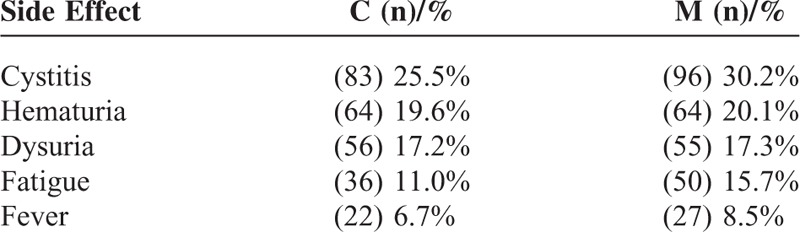
Summary Data for Side Effects

### Qualitative Risk of Bias and Quality Assessment

**FIGURE 8:**
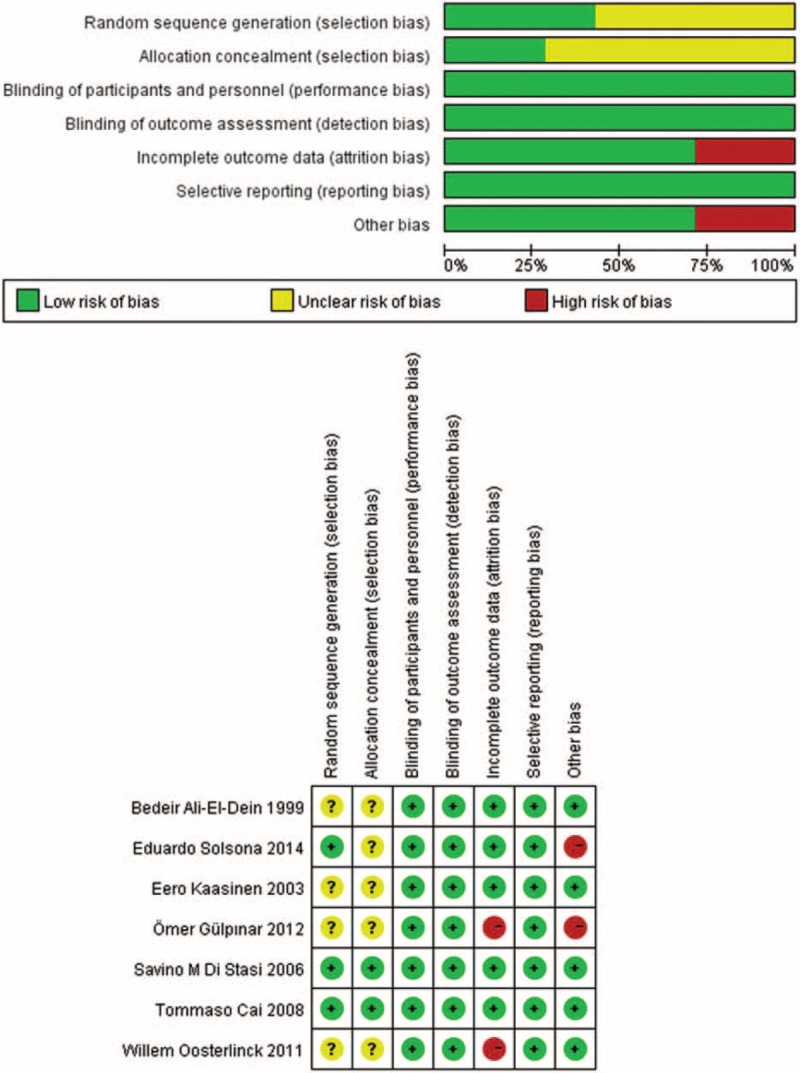
Graphic of the risk of bias assessment.

The quality of RCTs according to the Cochrane Collaboration handbook provided a qualification of the risk of bias. The results were shown in Figure 8. Three studies^15,16,20^ described the method of randomization, and 2^17,20^ described the allocation concealment; although only 1 study^20^ used the double-blind method. We defined the blind measurement of all studies as low risk of bias because the outcome measures in this meta-analysis were all objective. No selective outcomes were reported. Two studies^15,19^ did not include maintenance therapy, which was considered to be a high risk of other bias. Two studies were at low risk of bias, 2 studies were at moderate risk of bias, and 3 studies were at high risk of bias.

Our study had 3 outcome indicators. Disease-free survival was measured in all included studies, including 2 studies that explored the influence of a perioperative single dose instillation on delayed BCG therapy. Progression-free survival was measured in 5 studies. The GRADE system evidence for each outcome measure and the reasons for upgrade and downgrade were shown in Table 3.

**TABLE 3:**
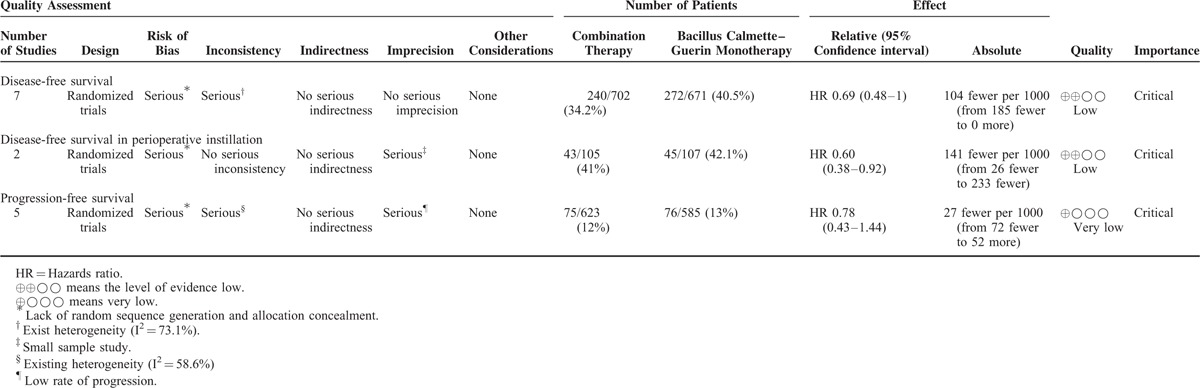
Grading of recommendation, assessment, development, and evaluation Profile Evidence of 3 Outcome Measures

### Publication Bias

**FIGURE 9:**
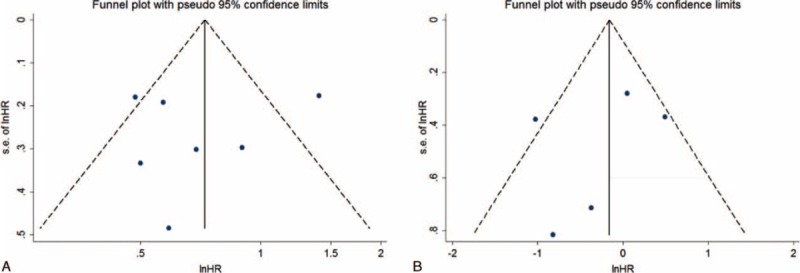
Funnel plot assessing publication bias: (A) disease-free survival (B) progression-free survival.

Funnel plot showed publication bias existed in DFS (Figure 9), but Egger test shows *P* = 0.695, so it maybe not caused by publication bias. So we made a sensitivity test. Nothing bias found in sensitivity test. The result showed no publication bias found by trim and fill method. The bias could origin from tumor stage and chemotherapy agent etc. No evidence of publication bias was found in PFS both funnel plot (Figure 9) by Egger test (*P* = 0.555).

## DISCUSSION

In the present meta-analysis, we identified a positive effect of combination therapy on DFS. In addition, perioperative single dose instillation could improve the efficacy of delayed BCG monotherapy in terms of DFS. Combination therapy, however, failed to show any significant difference from BCG monotherapy in terms of PFS. These results can help us make more precise recommendations for clinical practice.

It is important to note the therapy schedules in different studies because they can affect the results. Since Morales^4^ described a schedule of 6 weekly BCG instillations as the gold standard in 1976, all studies^15–21^ have used this induction period. Di stasi et al^16^ showed the great power of combination therapy, possibly because of the use of electromotive MMC and different instillation therapies: 1 MMC infusion was included after 2 BCG instillations during the induction period, and 2 MMC infusions were included after 1 BCG instillation in the maintenance period. Kaasinen et al^17^ and Oosterlinck et al^18^ showed different outcomes in patients with CIS alone. The European Organization for Research on Treatment of Cancer genitourinary group suggested that administering BCG instillation every 2 months in the alternating arm was suboptimal for the production of an adequate response. Thus, the results in these 2 therapy schedules need to be carefully discussed. Carcinoma in situ is high-risk tumor in bladder cancer, equal to T1 stage or G3 grade, so it is an important point in bladder cancer. There is still no consensus opinion concerning whether intravesical instillation or cystectomy should be performed for CIS treatment. Kaasinen et al^17^ suggested that the CIS category seemed to dictate the disease outcome. The currently accepted standard dose of BCG is 81 mg, as same as 5 × 10^8^ CFU, Kaasinen et al^17^ made BCG 120 mg instillation, it was out of the standard dose. And monthly instillation after induction period was different with Oosterlinck et al,^18^ which made another 6 weeks instillation after induction period. Also, Kaasinen et al^17^ got 1 year instillation, which could be another potential point to influence the results.

The heterogeneity in DFS and PFS could not be neglected. Owing to heterogeneous nature of the treatment schedules between studies, we chose random-effects models to evaluate the results directly. Meanwhile, we used different methods to search the origin of heterogeneity. Finally, we found different stages of tumor could explain the heterogeneity in DFS. We analyzed many potential reasons to explain heterogeneity in PFS, but only found electromotive MMC from Di Stasi et al^16^ was different from the others.

The primary outcome of this meta-analysis seemed to be different from a previous meta-analysis,^23^ which did not report in a significant reduction in recurrence. The potential reasons for this difference are as follows. Houghton et al^23^ used risk ratio to determine the effect size for dichotomous data and excluded studies that underwent less than 6 months of maintenance BCG treatment. Another study,^24^ however, showed that maintenance therapy did not result in a decrease in the recurrence and progression rates versus induction therapy alone. In an attempt to provide reliable results, our analysis used HR to determine the effect size. Remarkably, by using HR to determine the effect size and enrolling 3 new studies, we observed that combination therapy seemed to be associated with a longer DFS compared with BCG monotherapy. Furthermore, this meta-analysis evaluated the efficacy of different chemotherapy agents in combination therapy and found that epirubicin + BCG instillation improved DFS, whereas MMC + BCG instillation did not.

In a previous meta-analysis, Oosterlinck et al^25^ demonstrated that perioperative instillation reduced the relative risk of recurrence by 40% compared with transurethral resection of the bladder tumor alone; this meta-analysis evaluated the efficacy of a perioperative single dose instillation before delayed BCG monotherapy. One retrospective study^26^ was included in this meta-analysis; we excluded this study because it did not meet our criterion of all patients with intermediate- and high-risk NMIBC. Despite the exclusion of this study, the perioperative single dose instillation still improved the efficacy of delayed BCG monotherapy in DFS (HR = 0.60; 95% CI, 0.43–0.84; *P* = 0.003).

Both the previous meta-analysis^23^ and the present meta-analysis showed that combination therapy did not have an effect on PFS. Houghton^23^ found that combination therapy had a substantial effect in patients with Ta/T1 stage NMIBC, but no significant difference was found in this meta-analysis. Only receiving electromotive MMC had a potential effect on PFS. Di Stasi et al^27^ noted that intravesical electromotive delivery increased MMC transport and therefore its concentration in the tissue. This could represent a new method to improve the efficacy of combination therapy. Only 212 patients, however, were involved in the study. Therefore, the potential benefit should be discussed after further research.

Toxicity was an important consideration when formulating the therapy schedule. Because toxicity was hard to explain using HR to determine the effect size, we simply described the number and percentage of events. The number of local and systematic side-effects in the BCG monotherapy group was slightly higher compared with the combination therapy group, but this difference did not reach statistical significance.

Although evidence based on RCTs was considered to be high quality in the GRADE system at first, the level of outcome evidence could be decreased by 4 points by the following factors: lack of random sequence generation and allocation concealment; existing heterogeneity; studies with small sample sizes; and a low rate of progression. Thus, 2 studies had a low level of evidence and 1 was very low.

## LIMITATIONS

This study is restricted by the same problems that exist in most meta-analyses of drug trials: patients with different therapy schedules and follow-up times and different risks of recurrence and progression. It was difficult for us to obtain individual patient data, which is the gold standard for meta-analyses. Only studies in English were searched in this meta-analysis because other languages were outside of our ability.

Owing to the presence of significant heterogeneity in both DFS and PFS, we pooled the results using random-effects analysis, and made several subgroup analyses to explain the heterogeneity. Also, we used GRADE method to evaluate the strength of the result. The level of evidence was weak: 2 items were low, and 1 item was very low. Owing to the limited number of patients with progression, the PFS result could have imprecision bias. The efficacy in PFS could be more precise though large-scale RCTs. Although this meta-analysis suggested a significant benefit of 1 dose of perioperative chemotherapy, the result was suspect because of the limited number of patients.

These limitations may make the results unstable, so further studies are still needed to explore the gold standard of treating NMIBC.

## CONCLUSIONS

In conclusion, patients with NMIBC who underwent combination therapy had a benefit for prophylaxis against recurrence compared with those who underwent BCG therapy alone. No significant difference in PFS was found between combination therapy and BCG monotherapy. In addition, we identified the positive efficacy of 1 perioperative instillation compared with delayed BCG monotherapy. The positive result in our study was opposed to a previous result but was more reliable because of the use of HR to determine the effect size and the inclusion of a larger number of studies, particularly after using the conservative random-effects analysis.

## References

[1] Siegel RL Miller KD Jemal A Cancer statistics, 2015. *CA Cancer J Clin* 2015; 65:5–29. 25559415 10.3322/caac.21254

[2] Nieder AM Mackinnon JA Huang Y Florida bladder cancer trends 1981 to 2004: minimal progress in decreasing advanced disease. *J Urol* 2008; 179:491–495. 18076917 10.1016/j.juro.2007.09.082

[3] Brausi M Witjes JA Lamm D A review of current guidelines and best practice recommendations for the management of nonmuscle invasive bladder cancer by the International Bladder Cancer Group. *J Urol* 2011; 186:2158–2167. 22014799 10.1016/j.juro.2011.07.076

[4] Morales A Eidinger D Bruce AW Intracavitary Bacillus Calmette–Guerin in the treatment of superficial bladder tumors. *J Urol* 1976; 116:180–183. 820877 10.1016/s0022-5347(17)58737-6

[5] Duchek M Johansson R Jahnson S Bacillus Calmette–Guérin is superior to a combination of epirubicin and interferon-α2b in the intravesical treatment of patients with stage T1 urinary bladder cancer. A prospective, randomized, Nordic study. *Eur Urol* 2010; 57:25–31. 19819617 10.1016/j.eururo.2009.09.038

[6] Järvinen R Kaasinen E Sankila A Long-term efficacy of maintenance Bacillus Calmette–Guérin versus maintenance mitomycin C instillation therapy in frequently recurrent Tat1 tumours without carcinoma in situ: a subgroup analysis of the prospective, randomised FinnBladder I study with a 20-year follow-up. *Eur Urol* 2009; 56:260–265. 19395154 10.1016/j.eururo.2009.04.009

[7] Sylvester RJ Brausi MA Kirkels WJ Long-term efficacy results of EORTC genito-urinary group randomized phase 3 study 30911 comparing intravesical instillations of epirubicin, Bacillus Calmette–Guérin, and Bacillus Calmette–Guérin plus isoniazid in patients with intermediate- and high-risk stage Ta T1 urothelial carcinoma of the bladder. *Eur Urol* 2010; 57:766–773. 20034729 10.1016/j.eururo.2009.12.024 PMC2889174

[8] Babjuk M Burger M Zigeuner R EAU Guidelines on non–muscle-invasive urothelial carcinoma of the bladder: update 2013. *Eur Urol* 2013; 64:639–653. 23827737 10.1016/j.eururo.2013.06.003

[9] Parmar MK Torri V Stewart L Extracting summary statistics to perform meta-analyses of the published literature for survival endpoints. *Stat Med* 1998; 17:2815–2834. 9921604 10.1002/(sici)1097-0258(19981230)17:24<2815::aid-sim110>3.0.co;2-8

[10] Williamson PR Smith CT Hutton JL Aggregate data meta-analysis with time-to-event outcomes. *Stat Med* 2002; 21:3337–3351. 12407676 10.1002/sim.1303

[11] Tierney JF Stewart LA Ghersi D Practical methods for incorporating summary time-to-event data into meta-analysis. *Trials* 2007; 8:16. 17555582 10.1186/1745-6215-8-16 PMC1920534

[12] DerSimonian R Kacker R Random-effects model for meta-analysis of clinical trials: an update. *Contemp Clin Trials* 2007; 28:105–114. 16807131 10.1016/j.cct.2006.04.004

[13] Higgins JP Green S Cochrane Handbook for Systematic Reviews of Interventions Version 5.1.0 [updated March 2011]. In: Higgens JPT, Green LA, eds: The Cochrane Collaboration; 2011 http://handbook.cochrane.org/.

[14] Guyatt GH Oxman AD Vist GE GRADE: an emerging consensus on rating quality of evidence and strength of recommendations. *Br Med J* 2008; 336:924–926. 18436948 10.1136/bmj.39489.470347.AD PMC2335261

[15] E Madero R Chantada V Sequential combination of mitomycin C plus bacillus Calmette–Guerin (BCG) is more effective but more toxic than BCG alone in patients with nonmuscle-invasive bladder cancer in intermediate- and high-risk patients: final outcome of CUETO 93009, a randomized prospective trial. *Eur Urol* 2015; 67:508–516. 25301758 10.1016/j.eururo.2014.09.026

[16] Di Stasi SM Giannantoni A Giurioli A Sequential BCG and electromotive mitomycin versus BCG alone for high-risk superficial bladder cancer: a randomised controlled trial. *Lancet Oncol* 2006; 7:43–51. 16389183 10.1016/S1470-2045(05)70472-1

[17] Kaasinen E Wijkström H Malmström P Alternating mitomycin C and BCG Instillations versus BCG alone in treatment of carcinoma in situ of the urinary bladder: a Nordic study. *Eur Urol* 2003; 43:637–645. 12767365 10.1016/s0302-2838(03)00140-4

[18] Oosterlinck W Kirkali Z Sylvester R Sequential intravesical combination therapy with mitomycin C and bacillus Calmette–Guérin and with bacillus Calmette–Guérin alone in patients with carcinoma in situ of the urinary bladder: results of an EORTC genito-urinary group randomized phase 2 trial (30993). *Eur Urol* 2011; 59:438–446. 21156335 10.1016/j.eururo.2010.11.038

[19] Gulpinar O Halilioglu AH Gokce MI The value of perioperative mitomycin C instillation in improving subsequent bacillus Calmette–Guerin instillation efficacy in intermediate and high-risk patients with nonmuscle invasive bladder cancer: a prospective randomized study. *Int Braz J Urol* 2012; 38:474–479. 22951160 10.1590/s1677-55382012000400006

[20] Cai T Nesi G Tinacci G Can early single dose instillation of epirubicin improve bacillus Calmette–Guerin efficacy in patients with nonmuscle invasive high risk bladder cancer? Results from a prospective, randomized, double-blind controlled study. *J Urol* 2008; 180:110–115. 18485394 10.1016/j.juro.2008.03.038

[21] Ali-El-Dein B Ismail ANE Sequential bacillus Calmette–Guerin and epirubicin versus bacillus Calmette–Guerin alone for superficial bladder tumors: a randomized prospective study. *J Urol* 1999; 57:1310–1316. 10411034

[22] Colombel M Saint F Chopin D The effect of ofloxacin on bacillus Calmette–Guerin induced toxicity in patients with superficial bladder cancer: results of a randomized, prospective, double-blind, placebo controlled, multicenter study. *J Urol* 2006; 176:935–939. 16890660 10.1016/j.juro.2006.04.104

[23] Houghton BB Chalasani V Hayne D Intravesical chemotherapy plus bacille Calmette–Guérin in nonmuscle invasive bladder cancer: a systematic review with meta-analysis. *Br J Urol Int* 2012; 111:977–983. 10.1111/j.1464-410X.2012.11390.x 23253618

[24] Martinez-Pineiro L Portillo JA Fernandez JM Maintenance therapy with 3-monthly bacillus Calmette–Guerin for 3 years is not superior to standard induction therapy in high-risk nonmuscle-invasive urothelial bladder carcinoma: final results of randomised CUETO Study 98013. *Eur Urol* 2015; 68:256–262. 25794457 10.1016/j.eururo.2015.02.040

[25] Oosterlinck W Lobel B Jackse G EAU Recommendations 2001. “Guidelines on bladder cancer”. *Prog Urol* 2002; 12:1161–1163. 12536941

[26] Badalato GM Hruby G Razmjoo M Maximizing intravesical therapy options: is there an advantage to the administration of perioperative mitomycin C prior to an induction course of BCG? *Can J Urol* 2011; 18:5890–5895. 22018151

[27] Di Stasi SM Giannantoni A Stephen RL Intravesical electromotive mitomycin C versus passive transport mitomycin C for high risk superficial bladder cancer: a prospective randomized study. *J Urol* 2003; 170:777–782. 12913696 10.1097/01.ju.0000080568.91703.18

